# Spontaneous restoration of transplantation tolerance after acute rejection

**DOI:** 10.1038/ncomms8566

**Published:** 2015-07-07

**Authors:** Michelle L. Miller, Melvin D. Daniels, Tongmin Wang, Jianjun Chen, James Young, Jing Xu, Ying Wang, Dengping Yin, Vinh Vu, Aliya N. Husain, Maria-Luisa Alegre, Anita S. Chong

**Affiliations:** 1 Section of Rheumatology, Department of Medicine, The University of Chicago, 924 E. 57th Street, JFK-R302, Chicago, Illinois 60637, USA; 2 Section of Transplantation, Department of Surgery, The University of Chicago, 5841 S. Maryland Avenue, Chicago, Illinois 60637, USA; 3 Department of Biological Sciences, Chicago State University, 9501 S. King Drive, Chicago, Illinois 60628, USA; 4 Department of Pathology, University of Chicago, The University of Chicago, 5841 S. Maryland Avenue, Chicago, Illinois 60637, USA

## Abstract

Transplantation is a cure for end-stage organ failure but, in the absence of pharmacological immunosuppression, allogeneic organs are acutely rejected. Such rejection invariably results in allosensitization and accelerated rejection of secondary donor-matched grafts. Transplantation tolerance can be induced in animals and a subset of humans, and enables long-term acceptance of allografts without maintenance immunosuppression. However, graft rejection can occur long after a state of transplantation tolerance has been acquired. When such an allograft is rejected, it has been assumed that the same rules of allosensitization apply as to non-tolerant hosts and that immunological tolerance is permanently lost. Using a mouse model of cardiac transplantation, we show that when *Listeria monocytogenes* infection precipitates acute rejection, thus abrogating transplantation tolerance, the donor-specific tolerant state re-emerges, allowing spontaneous acceptance of a donor-matched second transplant. These data demonstrate a setting in which the memory of allograft tolerance dominates over the memory of transplant rejection.

## Abstract (web summary)

An infection can break the immune tolerance of a transplanted organ, resulting in its rejection. Here the authors show that the immunological memory of transplantation tolerance dominates over the memory of allograft rejection, so that another organ transplanted later can be spontaneously accepted.

## Graphical abstract

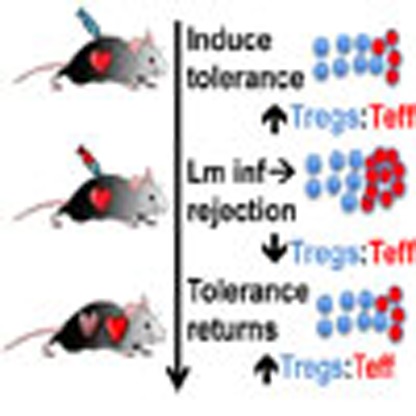

Solid organ transplantation is the therapy of last resort for end-stage organ failure, but, in the absence of immunosuppression, T-cell-dependent acute rejection of allografts invariably ensues. Rejection is accompanied by allosensitization and the accelerated rejection of a second donor-matched transplant, as first reported for humans and rabbits by Medawar and colleagues^1^,^2^. To prevent rejection, current immunosuppressive therapies that target T cells non-specifically have to be taken lifelong, leaving patients more susceptible to infections and tumours, in addition to having off-target side-effects. Donor-specific transplantation tolerance, in which alloreactive T cells are specifically incapacitated while leaving the rest of the immune responses intact, has long been the goal for clinical transplantation. Robust peripheral tolerance to allografts can be achieved in mice by administration of anti-CD154 (anti-CD40L) monoclonal antibody and donor-specific transfusion (DST)^3^. Such treatment at the time of transplantation results in long-term acceptance of a first cardiac allograft and subsequent acceptance of a second donor-matched heart, while allowing normal rejection of a second genetically distinct heart^4^.

In humans, transplantation tolerance has been challenging to achieve, but in recent years several groups have been able to induce it prospectively both in human leukocyte antigen (HLA)-matched and HLA-mismatched donor–recipient combinations^5^,^6^,^7^. In addition, a proportion of liver and renal transplant recipients treated with conventional immunosuppression and who subsequently discontinued treatment, achieved a state of operational tolerance in which the transplanted organ remained stably functional for years with minimal histological signs of graft pathology^8^,^9^,^10^,^11^. Notably, some of these tolerated transplants eventually succumbed to rejection, which manifested as a slow deterioration in graft function^12^. The underlying basis for allograft rejection after long periods of operational tolerance is not known, although in some instances bacterial infections have been described to precede graft loss^12^,^13^. These observations of a potential link between infection and graft loss are reminiscent of our previous report showing, in mice, that infection with the intracellular Gram-positive bacillus *Listeria monocytogenes* (Lm) ≥60 days after stable heart allograft tolerance precipitated rejection in a fraction of infected hosts^14^. Lm-triggered rejection was T-cell dependent, correlated with increased intragraft donor-specific T-cell alloreactivity in the absence of detectable crossreactivity between bacterial and donor antigens^15^, and was dependent on the production of interleukin (IL)-6 and signalling through the type I interferon receptor (IFNR)^14^. Furthermore, acute rejection of tolerant allografts in uninfected recipients was recapitulated by the combined induction of IL-6 and interferon (IFN)-β *in vivo*^14^.

It is assumed that the loss of operational tolerance in patients or animals is permanent such that re-challenge with a donor-matched graft would result in accelerated rejection, paralleling the consequences of acute rejection in a naïve host. Using a fully allogeneic cardiac allograft mouse model, we here report that the loss of transplantation tolerance following Lm infection-triggered allograft rejection is unexpectedly transient, as a second donor-matched transplant is spontaneously accepted after the temporary period of alloreactivity triggered by the infection waned. We show that this restored state of tolerance that dominates over a memory of rejection is mediated by regulatory T cells (Tregs) and exhibits exquisite specificity, as these recipients are able to generate protective immunity against the infectious agent and remain capable of rejecting third-party cardiac allografts.

## Results

### Return of tolerance after infection-induced graft rejection

Naïve hosts who acutely reject an allograft after transplantation develop allosensitization that results in the accelerated rejection of second donor-matched allografts transplanted at distal locations^1^,^2^,^16^. We tested whether tolerant hosts that reject an allograft following a bacterial infection become similarly allosensitized. To this end, we used an experimental model of cardiac transplantation, where fully mismatched BALB/c (H-2^d^) grafts vascularized in the abdominal cavity were accepted long term by C57BL/6 (H-2^b^) recipients following a transient treatment at the time of transplantation with anti-CD154 and DST. Intraperitoneal (i.p.) infection with Lm 60 days post transplantation overcame tolerance and induced a T-cell-dependent rejection in approximately half of the recipients (Supplementary Fig. 1a and ref. 14). This rejection was unlikely to be due to non-tolerant anti-Lm T cells that crossreacted with BALB/c antigens, as untransplanted C57BL/6 mice that had been pre-sensitized with Lm did not display enhanced responsiveness to BALB/c stimulators *in vitro* when compared with unsensitized controls (Supplementary Fig. 1b), or increased cytotoxicity to BALB/c targets *in vivo*^15^, or acquired resistance to costimulation blockade upon subsequent transplantation with BALB/c hearts in contrast to BALB/c-sensitized recipients^15^. We further confirmed that the rejection-triggering effect of Lm was not limited to the BALB/c to C57BL/6 strain combination, as a subset of C57BL/6 mice made tolerant to C3H/HEN (H-2^k^) cardiac allografts also rejected their transplant acutely following Lm infection (Supplementary Fig. 1c).

**Figure 1:**
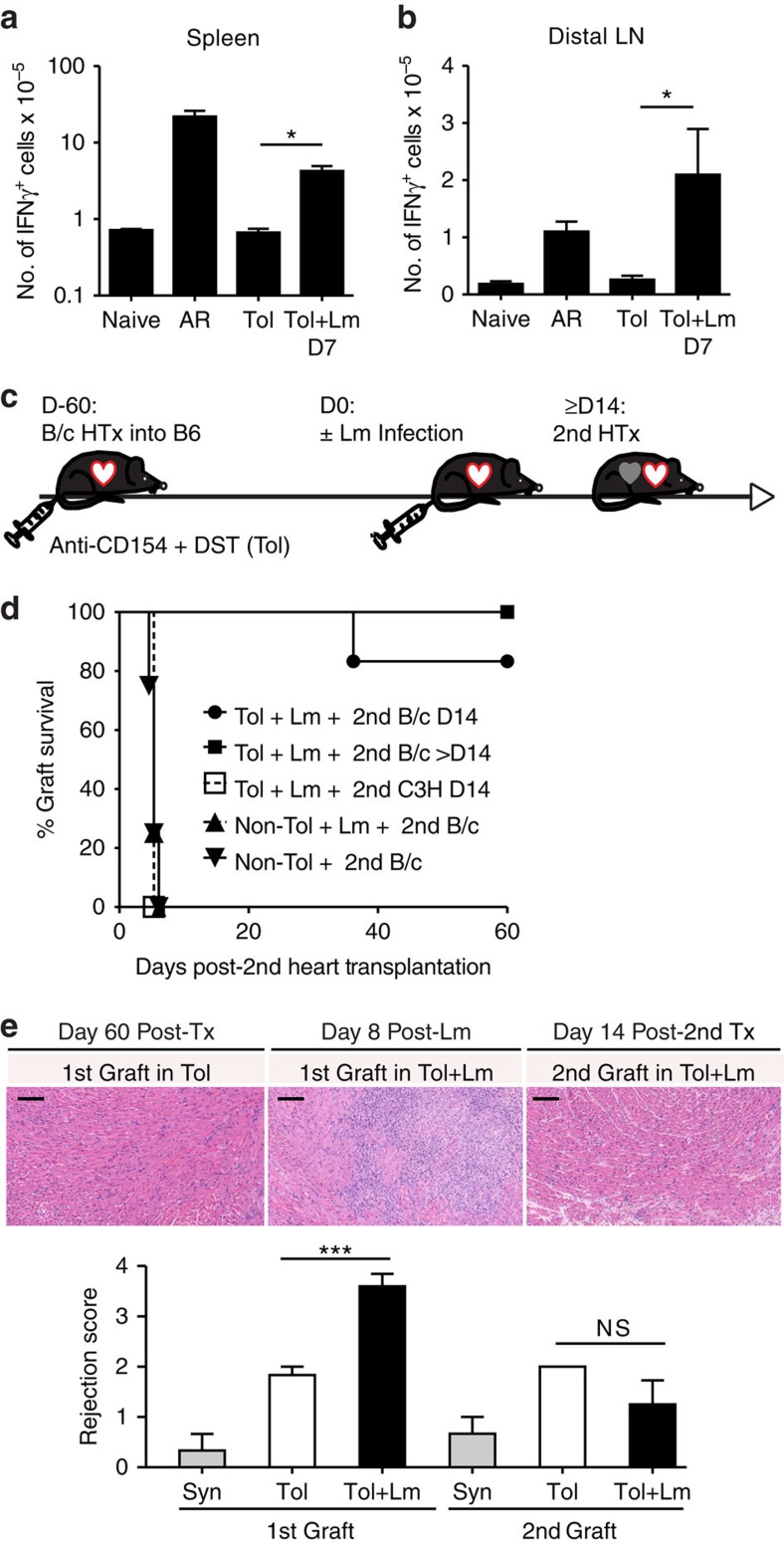
Absence of memory of transplant rejection following acute rejection of tolerated allografts. (**a**,**b**) BALB/c (B/c) hearts were transplanted into C57BL/6 (B6) recipients untreated (acute rejection (AR) group) or treated with anti-CD154+DST at the time of transplantation (tolerant (Tol) group). On day 60 (D60) post heart transplantation (HTx), a subset of tolerant mice were infected with Lm i.p. (Tol+Lm group). Untransplanted mice (naïve group) were used as controls. Splenocytes (**a**) and distal peripheral lymph nodes (distal LN) including cervical, brachial and axillary populations (**b**) from naïve, AR (D7 post HTx), Tol (D60 post HTx) and Tol+Lm (D7 post Lm) mice were stimulated with T-depleted B6 (syngeneic) or B6xB/c F1 (allogeneic) splenocytes. IFNγ-producing cells were analysed by intracellular flow cytometry on CD44^+^CD90^+^-gated events (naïve, Tol, Tol+Lm *n*=8 per group, AR *n*=4, experiment repeated twice, results pooled). The percentage of T cells specific for alloantigen (%allo–%syn) was multiplied by the number of live cells counted by the Trypan blue exclusion method to obtain the total number of IFNγ^+^ alloreactive T cells. Statistical significance at *P<0.05 by Kruskal–Wallis test with Dunn’s. (**c**) Experimental design for the transplantation of second allografts. (**d**) B6 mice treated with anti-CD154+DST at the time of B/c abdominal heart transplantation (on day minus 60), and that had rejected their allografts following Lm infection (3–5 × 10^6^ CFU i.p. on day 0 (D0); Tol+Lm) were re-transplanted in the neck on day 14 (D14) with B/c (*n*=6) or C3H (*n*=4) allografts, or on days >14 (21–42) with B/c allografts (*n*=4). As controls, animals transplanted with B/c hearts±Lm infection at the time of transplantation (non-Tol ±Lm+second B/c) received a second B/c heart 2 weeks later (*n*=4 each) in the neck. (**e**) Histology of 1st Tol B/c graft 60 days post transplantation (left), 1st Tol+Lm B/c graft at the time of rejection 8 days post infection (middle), and second B/c cervical graft D14 post second transplantation into mice that had rejected their first tolerated B/c heart following Lm infection (Tol+Lm, right), and composite histological scores. Scale bars, 100 μm. Data are presented as mean±s.e.m. of 3–6 hearts per group, repeated at least twice. Statistical significance by Student’s *t*-test at ***P≤0.001 or not significant (NS).

Consistent with allosensitization, we detected an increase in the number of IFNγ-producing alloreactive T cells at the time of graft rejection in the spleens of tolerant mice following i.p. Lm infection. We similarly observed an increase in the number of IFNγ-producing alloreactive T cells in peripheral lymph nodes (Fig. 1a,b) distal to the secondary lymphoid organs where T cells are primed following cardiac transplantation^17^. The presence of alloreactive T cells in both the spleen and distal lymph nodes supports their systemic recirculation from the sites of T-cell priming during the process of Lm-mediated loss of tolerance.

To assess whether this infection-triggered allograft rejection and increase in alloreactivity in formerly tolerant recipients would result in accelerated rejection of a second transplant, animals that had completely rejected their abdominal allografts following Lm infection were re-transplanted in the cervical area with either donor-matched (BALB/c) or third-party (C3H/HEN) cardiac allografts 14 days after Lm infection (Fig. 1c). Instead of undergoing rejection in an accelerated fashion, the second donor-matched hearts were spontaneously accepted long term in the absence of immunosuppressive therapy, whereas third-party C3H/HEN transplants were promptly rejected (Fig. 1d). Similar spontaneous acceptance of second BALB/c hearts was observed with grafts transplanted on day 42 rather than day 14 post infection of tolerant recipients to allow more time for potential memory responses to develop. Histology of second donor-matched cervical hearts at day 14 post transplantation confirmed an absence of acute cellular rejection, with similar low rejection scores in second hearts transplanted into previously infected or uninfected tolerant recipients and in contrast to high scores in the first hearts undergoing Lm-dependent acute rejection (Fig. 1e). Thus, unlike naïve animals that acutely reject second distal donor-matched allografts, tolerant animals that undergo infection-triggered acute rejection spontaneously accepted second distal donor-matched allografts.

### Donor reactivity following Lm infection is transient

**Figure 2:**
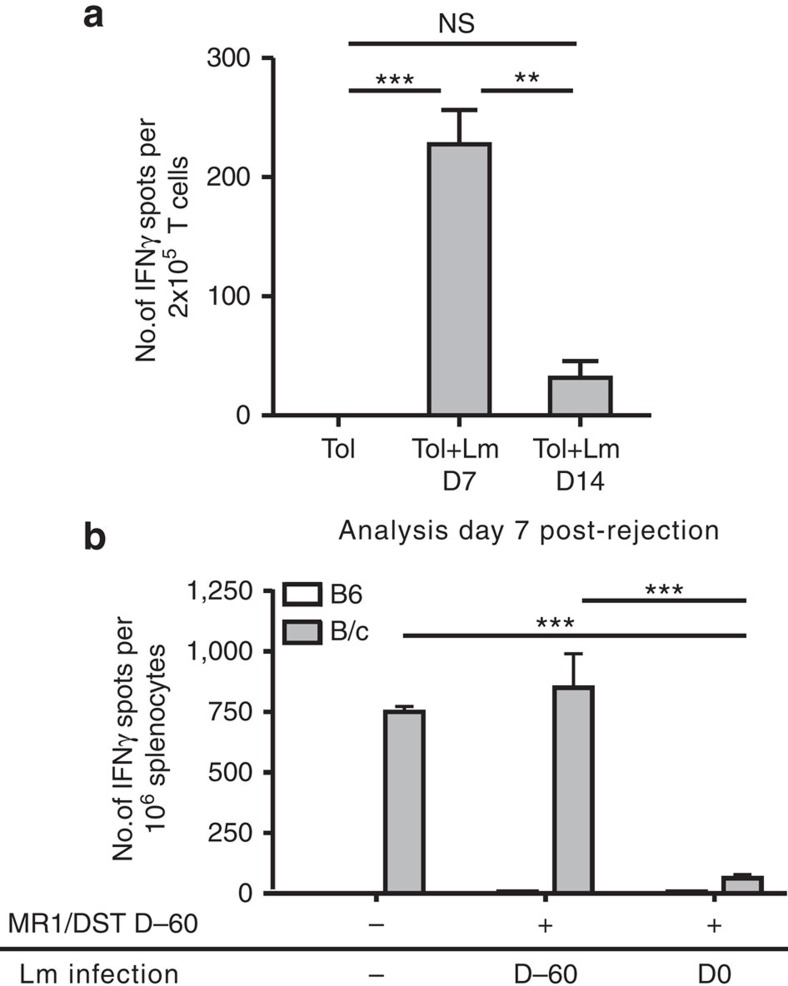
The detection of allosensitization after Lm-induced loss of tolerance is transient. (**a**) Splenic enriched T-cell populations from Tol+Lm mice were stimulated with T-depleted B6xB/c F1 and syngeneic B6 splenocytes, and were analysed by IFNγ ELISpot (*n*=3 per group, experiment repeated twice; syngeneic response was subtracted from allogeneic response). (**b**) Frequencies of alloreactive IFNγ-producing cells analysed by IFNγ ELISpot on day 7 (D7) after unmodified acute rejection or rejection in anti-CD154 (MR1)+DST-treated recipients infected with Lm on the day of transplantation (Lm D−60), versus following Lm-mediated rejection in tolerant (Tol) mice (Lm D0). *N*=3–5 per group and experiments repeated at least twice. Data are presented as mean±s.e.m. *P<0.05, **P<0.01, ***P<0.001; NS, not significant by one-way analysis of variance with Bonferroni or Kruskal–Wallis with Dunn’s for multiple comparisons.

To investigate the mechanism underlying the spontaneous acceptance of a second donor-matched allograft, anti-donor responses were measured at the time of second transplantation. Whereas the frequency (Fig. 2a and Supplementary Fig. 2) of detected IFN-γ-producing splenic alloreactive T cells was increased at day 7 post-Lm infection, coincident with the rejection of the established first allograft, this frequency was significantly reduced at the time of transplantation of the second allograft (14 days after Lm infection and 7 days after rejection of the established grafts) and was comparable to tolerant non-infected recipients. This was different from non-tolerant mice that had acutely rejected their grafts, and where significantly increased frequencies of IFNγ-producing alloreactive cells remained detectable 7 days after rejection (Fig. 2b). Furthermore, when Lm infection was on the day of transplantation, a time at which it prevents the induction of tolerance by anti-CD154/DST and triggers rejection 7–8 days after transplantation^15^, elevated frequencies of IFNγ-producing alloreactive T cells were also observed 7 days after acute rejection (Fig. 2b), indicating that Lm infection *per se* does not trigger the rapid reduction in the frequency of IFNγ-producing alloreactive T cells.

### The return of transplantation tolerance is Treg dependent

**Figure 3:**
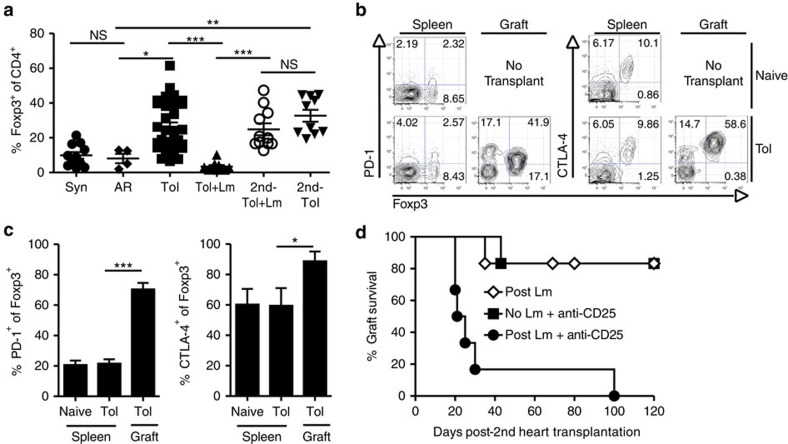
The return of tolerance is dependent on Tregs. (**a**) Cardiac allografts were harvested >60 days post transplantation for syngeneic grafts (Syn, *n*=11) or allogeneic tolerated grafts (Tol, *n*=34). Acute rejection (AR) allografts (*n*=4) were harvested 1 week post transplantation. Loss of tolerance hearts were harvested 1 week post Lm (with infection at 60 days post transplant; Tol+Lm, *n*=10). For the restoration of tolerance, second donor cardiac allografts were transplanted 14 days after Lm infection of tolerant recipients, that is, 1 week post rejection of the first allograft (second-Tol+Lm, *n*=11). Control second donor allografts were transplanted in tolerant non-infected mice (second-Tol, *n*=10) at a comparable time (74 days post first heart transplant). Second grafts were harvested 2 weeks after transplantation. Graft-infiltrating leukocytes were analysed by flow cytometry, gated on CD4^+^ events and displayed as % Foxp3^+^ of CD4^+^ cells. Data are presented as mean±s.e.m. and are pooled from 2 to 17 replicate experiments. (**b**,**c**) Representative flow cytometry plots of PD-1 and CTLA-4 expression on Foxp3^+^ Tregs in the spleen and grafts of naïve, untransplanted and tolerant mice (**b**) and quantification (**c**). Naïve spleen *n*=12, Tol spleen *n*=9, Tol graft *n*=12. Data are presented as mean±s.e.m. and are pooled from 7 to 8 experiments. *P<0.05, **P<0.01, ***P<0.001; NS, not significant by one-way analysis of variance with Bonferroni or Kruskal–Wallis with Dunn’s for multiple comparisons. (**d**) Fourteen days after Lm infection, recipients with rejected hearts and uninfected tolerant mice were transplanted with donor-matched heart grafts. Anti-CD25 treatment was administered once, the day before second heart transplantation (*n*=6 each). Controls (post Lm, *n*=6) are the same as shown in Fig. 1d. Data are presented as percent graft survival of the second B/c hearts with or without anti-CD25 therapy (*P*<0.01 by log-rank test) and are pooled from three experiments.

**Figure 4:**
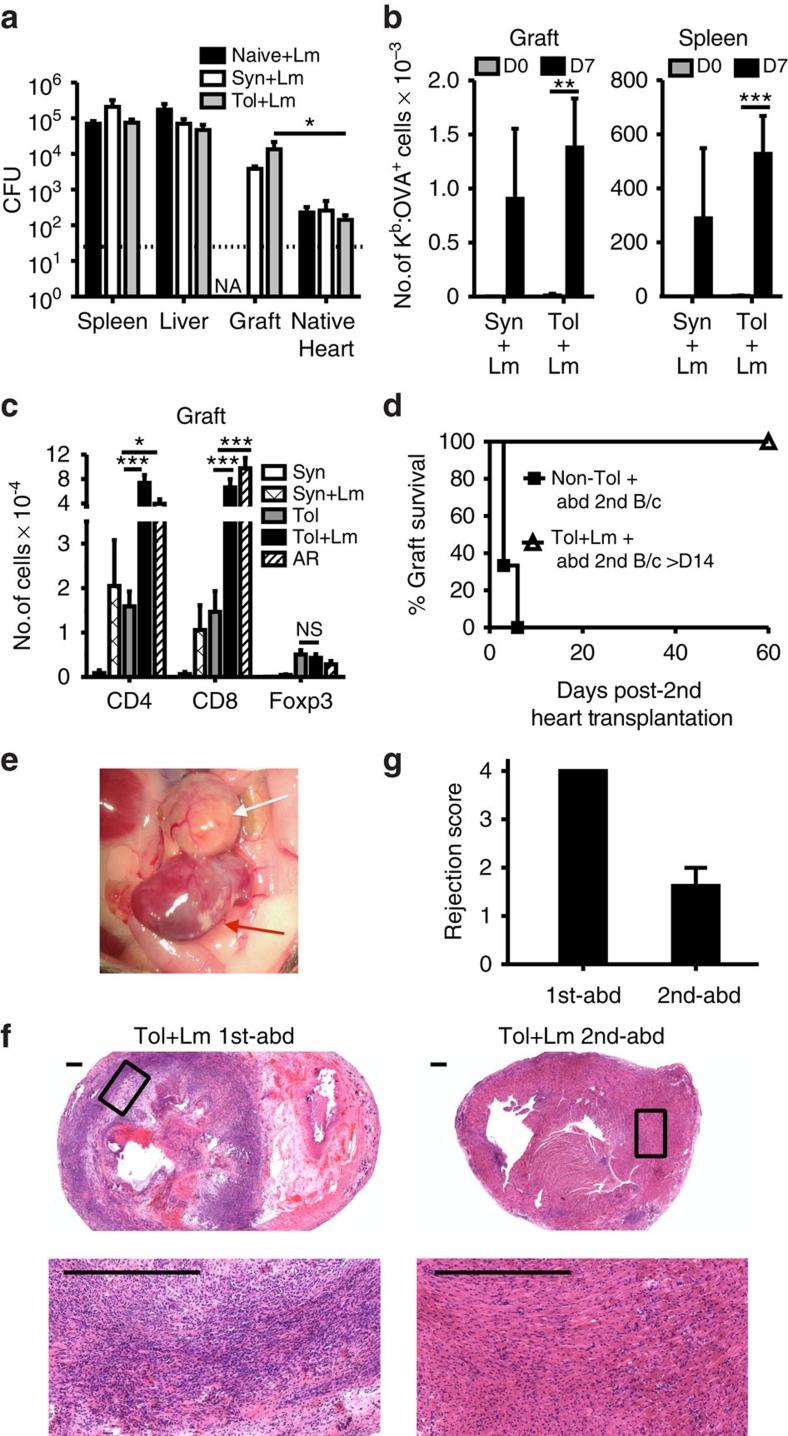
Lm infection elicits a systemic alloresponse in tolerant mice. (**a**) CFU numbers at 48 h post-Lm infection in the spleen, liver, grafts and native hearts of B6 mice that were either not transplanted (Naïve), or were tolerant to an allogeneic B/c heart (Tol) or had received a syngeneic heart (Syn) at least 60 days before Lm infection. Results are pooled from two independent experiments: naïve+Lm (*n*=7), Tol+Lm (*n*=10), Syn+Lm (*n*=3). NA, not applicable. *P<0.05 by Kruskal–Wallis with Dunn’s post test. (**b**) The total number of K^b^:OVA-binding Lm-specific T cells in the graft and spleen of syngeneic (Syn) or tolerant (Tol) mice, on D0 or D7 post infection, was determined by flow cytometry. Syn D0 *n*=2, Syn+Lm D7 *n*=4, Tol D0 *n*=4, Tol+Lm D7 *n*=5. Data are pooled from two independent experiments. **P<0.01 or ***P<0.001 by two-way analysis of variance (ANOVA) with Bonferroni post test. (**c**) Total numbers of CD4^+^, CD8^+^ and CD4^+^ Foxp3^+^ T cells within syngeneic (Syn) or tolerated (Tol) grafts, or 7–8 days post-Lm infection, and in grafts from untreated mice undergoing AR (data pooled from three independent experiments. Syn *n*=2, Syn+Lm *n*=4, Tol *n*=8, Tol+Lm *n*=8 and AR *n*=4). *P<0.05 or ***P<0.001 by two-way ANOVA with Bonferroni post test. (**d**) Tol+Lm mice that had rejected their allografts following Lm infection (3–5 × 10^6^ CFU i.p. on D0) were re-transplanted in the same location (abdominal area) on days 19–21 post Lm with B/c allografts (abd *n*=5). AR mice (non-Tol) were re-transplanted in the abdominal area on days 21–34, (*n*=3, *P*<0.01 by log-rank test). (**e**) Example (from **d**) of a white fibrotic rejected first allograft in a Tol+Lm mouse (white arrow), and of the second well-vascularized beating allograft transplanted immediately below the rejected allograft 3 weeks after Lm infection, in the absence of immunosuppression (red arrow; Supplementary Movie 1). The photograph was taken at D60 after second transplantation. (**f**) Representative histology of first abdominal B/c graft (Tol+Lm 1st abd, left) and second B/c abdominal graft (Tol+Lm 2nd abd, right) of the mice described in (**d**,**e**) taken at D60 post transplantation of the second allograft at lower (top) and higher (bottom) magnifications. Scale bars, 200 μm. (**g**) Composite histological scores (mean±s.e.m. of four hearts per group).

The transiency of the measurable alloresponse after Lm infection of tolerant mice could theoretically be due to impaired survival or function of the alloreactive T cells, and/or regulation by Foxp3^+^ T cells. Indeed, we had previously shown that tolerated allografts contained a high percentage of CD4^+^CD25^+^Foxp3^+^ regulatory T cells among CD4^+^ cells^14^,^18^. We confirmed here that the high percentage of CD4^+^Foxp3^+^ cells among CD4^+^ events in the first accepted allografts was significantly reduced during Lm-dependent acute rejection of the first allografts. This reduction in percentage of Foxp3^+^ cells was due to an influx of effector T cells as the total number of Tregs remained unchanged with Lm infection (ref. 14 and Fig. 4c). Of interest, the percentage of Foxp3^+^ cells was re-established in the donor-matched second allografts transplanted 14 days after Lm infection (Fig. 3a), and was in fact similar to the percentage of CD4^+^Foxp3^+^ T cells in second donor-matched allografts transplanted into non-infected tolerant recipients. This Treg percentage was significantly higher than that detected in syngeneic grafts, consistent with the tolerance to allografts being actively maintained by Tregs. Finally, a greater percentage of Tregs from tolerated allografts expressed a PD-1^high^ and CTLA-4^high^ phenotype of activated Tregs^19^, compared with Tregs from the spleen of naïve or tolerant animals (Fig. 3b,c).

To determine whether functional alloreactive T cells persisted at the time of second transplantation and whether they were suppressed by regulatory cells after the infection subsided, previously tolerant animals that had rejected their abdominal graft following Lm infection were depleted of Tregs by treatment with the anti-CD25 monoclonal antibody PC61 1 day before the transplantation of a second cervical BALB/c heart. Anti-CD25 administration prevented the acceptance of the second cervical transplants in animals that had rejected their first abdominal allografts after Lm infection (Fig. 3d). Thus, these data confirm that alloreactive T cells capable of rejecting a transplant had been induced following Lm infection, and persisted systemically at the time of second transplantation, but their function was dominantly suppressed by CD25^+^ regulatory cells. In contrast, anti-CD25 administration did not trigger rejection of second allografts transplanted into tolerant uninfected recipients.

### Local return of tolerance at the site of infection

Because Lm was injected i.p., it was likely that the bulk of the infection, inflammation and anti-Lm immune responses were occurring in the abdomen. This raised the possibility that Lm infection may have abrogated transplantation tolerance permanently in the peritoneal cavity where the first heart was located, whereas distal tolerance may have persisted to permit acceptance of the second hearts placed in the cervical area. I.p. Lm infection indeed resulted in high bacterial counts in the spleen and liver of untransplanted, syngeneic and allogeneic graft recipients, and in the syngeneic and allogeneic abdominal grafts, whereas significantly lower counts were detected in the native heart, outside of the peritoneal cavity (Fig. 4a). The infection was associated with a strong anti-Lm response in the graft and spleen of allograft recipients, as determined using Lm engineered to express the model antigen ovalbumin (OVA) and fluorescently-coupled K^b^:OVA pentamers to identify Lm-reactive T cells (Fig. 4b), and intragraft Lm-specific T cells contributed to the overall increase in the total number of effector T-cell subsets in the grafts (Fig. 4c). Furthermore similar Lm colony-forming units (CFUs) and anti-Lm T cells were detected in syngeneic cardiac grafts from infected mice (Fig. 4a,b), but only the allogeneic but not the syngeneic grafts stopped beating after Lm infection^14^. These observations therefore confirm that a strong local inflammatory response to Lm was elicited within the abdominal graft, even though it was not sufficient to trigger graft loss in the absence of alloreactivity.

To test whether transplantation tolerance was permanently abrogated at the site of the Lm infection, animals in which a primary abdominal allograft had completely stopped following Lm infection were re-transplanted 14–21 days post first allograft rejection with a donor-matched cardiac allograft placed also in the abdominal cavity, immediately adjacent to the primary rejected heart (rather than in the cervical area as shown in Fig. 1d). Remarkably, these second abdominal allografts were also spontaneously accepted (Fig. 4d–g, and see video of non-beating rejected first heart and beating accepted second heart side-by-side in Supplementary Movie 1). These data indicate that tolerance returned even at the site of maximal infection, thus underscoring the resilience of robust transplantation tolerance that could be transiently abrogated during an intense inflammatory response, but returned to dominate over alloreactivity when the infection was cleared.

### Development of memory to Lm in transplant-tolerant mice

**Figure 5:**
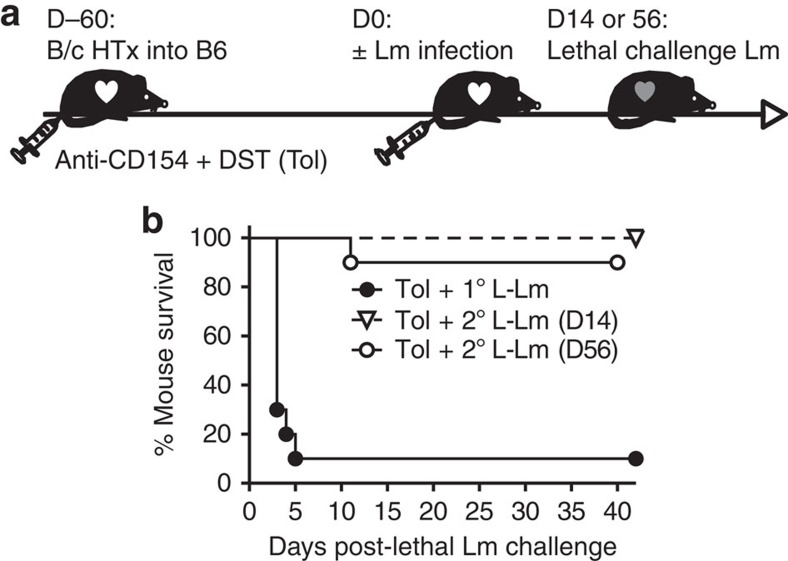
Animals experiencing a return of transplantation tolerance can successfully develop protective immunity to a lethal challenge of Lm. Tolerant (Tol) mice received a primary lethal (L) challenge of Lm (1.2 × 10^7^ CFU; 1° L-Lm, *n*=10) or a sublethal Lm infection on day 0 (D0) followed by graft rejection and subsequent re-infection with a secondary lethal Lm challenge (2° L-Lm) on D14 or D56 after the first infection (*n*=7–9 per group). Data are pooled from two independent experiments. (**a**) Experimental design. (**b**) Data are presented as percent animal survival after lethal Lm challenge (*P*<0.001 for 2° L-Lm on D14 or D56 compared with 1° L-Lm by log-rank test).

The inability of tolerant mice to demonstrate a functional memory of the transplant rejection prompted us to test whether this defect extended to the development of protective immunity to Lm. Lm-infected tolerant animals were challenged with a dose of Lm that was lethal to tolerant recipients that had not been previously infected (Fig. 5a). Memory to Lm developed successfully as the Lm-infected tolerant animals were protected against a lethal challenge of Lm administered either 2 or 8 weeks after the first infection (Fig. 5b). Thus, Lm infection abrogated established transplantation tolerance without inducing a functional allograft-specific memory, whereas anti-Lm memory developed normally.

## Discussion

We here provide experimental evidence of a transplant outcome that does not follow the rules of allosensitization described for non-tolerant animals by Medawar and colleagues^1^,^2^, and demonstrate in mice with established transplantation tolerance that a memory of regulation can dominate over a memory of infection-triggered rejection. We previously reported that the loss of transplantation tolerance following Lm infection depended on the production of the inflammatory cytokines IL-6 and IFNβ (ref. 14). We, and others, have shown that IL-6 augments effector T-cell responses, rendering them less susceptible to suppression by Tregs *in vitro* and preventing iTreg differentiation, whereas IFNβ promotes the acquisition by conventional T cells of IFNγ production and its consequent effector functions^14^,^20^,^21^. Our current results suggest that the pro-inflammatory effects of the Lm response are transient even at the site of infection, where the concentration of pro-inflammatory cytokines is presumed to be the highest. Finally, the spontaneous acceptance of second donor-matched allografts post Lm was prevented by anti-CD25 treatment, confirming the systemic presence of alloreactive T cells capable of driving rejection after Lm infection of tolerant mice, and implicating functional suppression by regulatory cells for tolerance to dominate over the memory of rejection.

The susceptibility to rejection following infection in recipients with established tolerance has implications for the notion that tolerance can achieve the goal of ‘one transplant for life’ in humans^22^. Indeed, viral as well as other types of bacterial infections have been described in immunosuppressed as well as tolerant transplant patients preceding episodes of acute rejection or allograft loss, especially when infections are in the vicinity of the graft^12^,^23^,^24^,^25^. Even though Lm infections have been reported in transplanted patients^26^, thereby providing a clinical context for our experimental model, it is important to point out that we used this single infectious agent in two donor–recipient combinations such that the generalizability of infections breaking established transplantation tolerance is not yet clear.

We speculate that infections close to or within the grafts may be more likely than distal infections to achieve local levels of inflammatory cytokines that are capable of uncoupling graft-infiltrating alloreactive T cells from the immunosuppressive pathways that keep them in check. We had previously demonstrated that the ability of Lm to break established transplantation tolerance is the result of synergy between the inflammatory axes of type I IFN and IL-6 signalling^14^. While cytokine production is likely to be dampened in transplant patients maintained on pharmacological immunosuppression, it might become more relevant and have more potent immunological consequences when protocols to induce clinical tolerance or minimize immunosuppression become more widespread. It is therefore important to define the spectrum of microbes and pro-inflammatory signals that are capable of enhancing alloreactivity and triggering a loss of tolerance.

Our observation that only a subset of tolerant infected mice fully reject their hearts is also similar to the clinical observation that not all infected patients reject their allografts^12^. The underlying basis for this variable susceptibility to rejection following infection remains to be fully investigated, but we hypothesize that stochastic differences in the strength of the immune response to infection and the robustness of tolerance in the recipients may be contributory factors.

The identification of a high percentage of Tregs in both primary tolerated and secondary ‘return of tolerance’ allografts, and their expression of an activated phenotype, as well as the dependence on Tregs for the return of tolerance following infection is reminiscent of previous reports of regulatory memory. Rosenblum *et al.*^19^ described memory Tregs in transgenic mice that expressed both OVA-specific DO11.10 T cells, and, upon doxycycline treatment, OVA in the skin. In those mice, T-cell-dependent dermatitis occurred following expression of OVA, and Tregs with an activated phenotype accumulated in the skin and limited secondary responses more potently than primary ones, suggesting that a response to a self-antigen can imprint regulatory memory in tissues. Similarly, Rowe *et al.*^27^ reported regulatory memory in the context of immune responses to fetal antigens. In that study, antigen-specific Tregs persisted after parturition and rapidly expanded during secondary pregnancy to more strongly suppress fetal resorption. Our results not only extend those observations to a transplantation setting but also demonstrate that regulatory memory can be therapeutically induced, and is migratory to a second allograft. More importantly, our data also establish a new paradigm for transplantation tolerance that regulatory tolerance can be transiently overcome by acute infections to allow allospecific effector responses to emerge, but that the tolerance can be re-established when the inflammatory signals recede with the clearance of the infection.

Regulatory T-cell suppression of persisting alloreactive T cells has been shown to be critical for the maintenance of skin allograft tolerance induced with a combination of anti-CD4, anti-CD8 and anti-CD154 antibodies^28^. In contrast, our observation that the maintenance of established tolerance to heart allografts in uninfected recipients is not dependent on CD25^+^ regulatory T cells is consistent with a previous report showing that anti-CD25 treatment prevented the induction of tolerance to cardiac allografts by anti-CD154, but did not abrogate tolerance after it had been established^29^. Because anti-CD154/DST treatment has been shown to also drive partial deletion and anergy of alloreactive T cells^30^,^31^, it is likely that multiple redundant mechanisms of tolerance cooperate to maintain cardiac allograft survival after anti-CD154/DST therapy in the uninfected recipient. In contrast, in post-infected tolerant recipients, depletion of CD25^+^ cells is sufficient to prevent the acceptance of secondary donor allografts. We reason that Lm may impair other pathways of tolerance induced by anti-CD154, namely, partial deletion and anergy of alloreactive T cells, but not the suppressive function of Tregs, or that Lm infection-mediated erosion of anergy or expansion of alloreactive T cells may be more durable than its effect on Tregs, thereby allowing Tregs to play a dominant role in the return of tolerance. Thus, depending on the mechanisms of maintenance of tolerance elicited by specific therapeutic regimens, the types of inflammatory pathways triggered by particular infections, and the duration of their impact, one could conceive that certain infections might have no significant impact on tolerance, whereas others might precipitate an irreversible loss of tolerance. The latter may be achieved by simultaneously permitting the expansion of effector T cells and permanently impairing regulatory T-cell maintenance or function. Indeed, chronic inflammation including inflammatory cytokines, such as IL-6 and TNF, has been shown to promote the instability of the Treg lineage and to impair the function of regulatory cells^32^.

While our studies point to a Treg-dependent return of tolerance, Schietinger *et al.*^33^ have described a model in which cell-intrinsic T-cell anergy was lost and spontaneously restored. In the study by Schietinger *et al.*^33^, CD8^+^ TCR transgenic T cells tolerant to a Friend murine leukaemia virus (FMuLV) GAG epitope expressed in hepatocytes transiently recovered effector function when these cells were adoptively transferred into lymphodeficient hosts lacking the GAG epitope, but then returned to an unresponsive state^33^. Whether similar cell-intrinsic unresponsiveness of alloantigen-specific T cells can also contribute to the maintenance and restoration of transplantation tolerance post infection, in addition to regulatory memory as we have shown, will be of interest to pursue in future studies.

The concept that tolerance can be overridden transiently during inflammatory events, but then re-surfaces when the inflammation resolves, may have wide clinical implications. It may explain why certain transplant patients can be successfully weaned of immunosuppression, revealing a state of operational tolerance despite having experienced prior acute rejection events^12^,^34^. Moreover, several autoimmune diseases are known to undergo phases of relapse and stages of remission. It is possible that disease relapse is triggered by pro-inflammatory events that overwhelm an already suboptimal self-tolerant state in individuals genetically predisposed to autoimmunity. With the quiescence of inflammation, regulation may dominate again to explain disease remission. Similarly, initial immune-dependent regression of tumours can be followed by tumour recurrence. Tumour elimination by antitumour T cells may be aided by bystander inflammation, while tumour recurrence may be facilitated by activated Tregs^35^. In fact, Lm is currently being used in clinical trials to improve antitumour immunity^36^, suggesting that similar mechanisms of tolerance in the context of cancer as in our transplant model may be overcome by Lm infection. Therefore, a better understanding of the spontaneous restoration of antigen-specific tolerance may have wide clinical applicability for therapeutic approaches to transplantation, autoimmunity and cancer.

## Methods

### Study design

To calculate the sample size for the comparison of means of two independent samples, utilizing the online sample size calculator from Rollin Brant (University of British Columbia), with at least *n*=3 per group, there is 80% power to detect 3.5-fold differences or greater even if the average s.d. is as large as the smaller group’s mean. With at least *n*=4 per group and *α*=0.05, there is 80% power to detect threefold differences or greater in population means. With these same parameters, to have 80% power to detect a 2.5-fold change, at least seven individuals per group were needed and to detect a twofold change, at least 16 individuals per group were needed.

If the s.d. is half the smaller group’s mean, then *n*=4 is sufficient at 80% power to see twofold changes or greater, and *n*=3 is sufficient at 80% power to see 2.25-fold changes or greater. When results were not Gaussian, non-parametric statistics were used in the final analysis with often twice the number of samples than would be required to see a difference if the data were Gaussian once multiple experiments are pooled. Multiple comparisons were corrected by Bonferroni (parametric) or Dunn’s (non-parametric) tests.

For graft survival analyses, using the PS programme (Power and Sample size version 3.1.2), with *α*=0.05, a follow-up time of 60 days and the control group rejecting with a mean survival time (MST)=7 days, an average of *n*=4 animals were needed per group to detect hazard ratio of 0.137 or lower. This shifts to *n*=5 for hazard ratios of 0.17 or below, and to *n*=6 for hazard ratios of 0.198 or below. These numbers remain similar if the shorter median survival time of two groups is 23 days, provided there is a longer follow-up time of 120 days. For animal survival analyses, the PS programme was also used. With *α*=0.05, a follow-up time of 20 days, and the primary lethal infection group succumbing to infection with a MST of 3 days, *n*=4 animals were needed per group to detect a hazard ratio of 0.164 or lower.

No outliers were excluded and each experiment was repeated at least twice. The number of replicate experiments and whether the results were pooled from multiple experiments is listed in each figure legend.

### Mouse transplantation and Lm infection

Six- to eight-week-old female C57BL/6 (B6, H-2^b^) mice as recipients and BALB/c (B/c, H-2^d^) and C3H/HEN (C3H, H-2^k^) mice as donors were purchased from Jackson or Harlan Laboratories. Mouse abdominal heterotopic cardiac transplantation was performed using a modified technique from Corry and colleagues^15^ in which the aorta and pulmonary artery of the graft end-to-side was anastomosed to the recipient’s aorta and vena cava, respectively, while cervical area heterotopic cardiac transplantation was performed with the aorta and pulmonary artery of the graft anastomosed end-to-end to the recipient’s common carotid artery and jugular vein, respectively. Transplantation of secondary hearts in the abdominal cavity was performed with vascular anastomoses similar to (aorta and pulmonary artery of the graft to the abdominal aorta and inferior vena cava of the host), but immediately adjacent (above or below) to, those of the primary allograft. The rejected primary allograft was left in place and recipients did not receive antibiotics. Tolerance was induced by administration of anti-CD154 (BioXCell; 0.3–0.5 mg per mouse, intravenous on day 0, i.p. on day 7 and day 14 post transplantation) and DST (10^7^ donor splenocytes on the day of transplantation) to recipient mice. Cardiac grafts were checked by palpation and the day of rejection was defined as the last day of detectable heartbeat. Lm engineered to express the model antigen OVA was grown overnight and then re-diluted 1:50 the next day and regrown for 1.5 h to obtain cultures in early log-phase growth before enumeration of CFU by measurement of the absorbance at OD_600_ with a spectrophotometer^15^. Doses of 3–5 × 10^6^ CFU (i.p.) were chosen for infection experiments to obtain highest rejection rate with minimal lethality. In some experiments, Lm (10^5^ CFU) was administered on the day of transplantation to mice that did not receive immunosuppression (acute rejection group) as a control. Allograft rejection was determined by heartbeat cessation using manual palpation of the closed abdomen. In addition, abdominal hearts were visually inspected before killing mice to ensure that the grafts never regained a heartbeat following Lm-dependent rejection. For lethal Lm challenge, mice were infected with 1.2 × 10^7^ CFU (i.p.). All Lm-infected mice were kept in biosafety facilities. All animals were used in agreement with the University of Chicago Institutional Animal Care and Use Committee, according to the National Institutes of Health guidelines for animal use.

### Histopathology

Grafts were removed and placed in 10% formalin. Sections were stained with haematoxylin and eosin, and the slides were scanned via CRi Pannoramic Scan Whole Slide Scanner at × 40, and then patched together to form one single image of the tissue. Pannoramic Viewer (a free downloadable software from 3DHISTECH) was used to view these image files. Images were examined by two independent investigators in a blinded manner. Histological sections were scored based on the percentage of infiltration observed in the interstitial tissue and as follows: 0%=0, 1–25% =1, 25–50%=2, 50–75%=3 and 75–100%=4.

### IFNγ ELISpot assays

T cells from spleen were purified by negative selection using cell isolation kits (Miltenyi Biotec Inc.), and cell purity was confirmed to be >93% by flow cytometry. T cells (2 × 10^5^ per well in triplicate) were co-cultured for 24 h with T-cell-depleted irradiated (3,000 rads) C57BL/6xBALB/c (F1) or BALB/c splenocytes, or with syngeneic (B6) splenocytes incubated with or without heat-killed Lm (5 × 10^5^ per well). An IFNγ ELISpot kit was used according to manufacturer’s instructions (BD Biosciences) and the numbers of spots per well were enumerated using the ImmunoSpot Analyzer (CTL Analyzers LLC).

### Isolation of graft-infiltrating cells

Graft-infiltrating cells were isolated from cardiac grafts that were extensively rinsed with heparin/1 × Hank's balanced salt solution (HBSS) solution (Cellgro), cut into small pieces and digested with 400 U ml^−1^ collagenase IV (Sigma), 0.01% DNase I (MP Biomedicals) and 10 mM HEPES (Cellgro) in HBSS. The cells were then washed, stained and analysed by flow cytometry.

### Enumeration of Listeria colony-forming units

Spleens, livers, transplanted hearts and native hearts were isolated from each animal at 48 h post-Lm infection. Tissues were homogenized first with a surgical blade and then with a Tissue Tearor hand-held homogenizer (Biospec Products) in 0.05% Tween in water. Serial dilutions of the homogenates were plated on Brain Heart Infusion agar (BD Biosciences) and incubated at 37 °C for 24 h before colonies were counted.

### Flow cytometry analysis

Splenocytes and total graft-infiltrating cells were stained using monoclonal antibodies specific for mouse CD90.2 (53-2.1, cat. no. 0902), CD4 (GK1.5, cat. no. 0041), CD8 (53-6.7, cat. no. 0081), CD44 (IM7, cat. no. 0441), PD-1 (J43, cat. no. 9985), CTLA-4 (UC10-4B9, cat. no. 1522), IFNγ (XMG1.2, cat. no. 7311) and Foxp3 (FJK-16 s, cat. no. 5773) (eBioscience), and in some experiments, K^b^:OVA pentamers (ProImmune) to identify Lm-OVA-specific T cells, and analysed by flow cytometry (LSRII, BD). Pentamer staining was performed using one test (10 μl) to stain 5 million cells in 100 μl. For intracellular cytokine analysis, whole-lymph node cells (cervical, axillary, brachial and inguinal) or splenocytes were stimulated for 24 h with T-cell-depleted BALB/c × C57BL/6 F1 or C57BL/6 splenocytes with or without heat-killed Lm, and all samples were incubated with GolgiPlug (BD Biosciences) for the last 6 h. Cells were resuspended at 10^6^ cells per 20 μl, and the following dilutions of 0.2 mg ml^−1^ of antibodies to CD90.2 (1:400), CD8 (1:100), CD4 (1:100), PD-1 (1:100), CTLA-4 (1:00) and IFNγ (1:100) were used. CD44 and Foxp3 antibodies were diluted 1:100 from a 0.5 mg ml^−1^ stock. Cells were fixed and permeabilized for intracellular staining using a Foxp3 buffer staining kit (eBioscience). Viability of the cells was determined by using Viability Dye (for fixed samples) or 4,6-diamidino-2-phenylindole (for unfixed samples; Invitrogen). Data were analysed using FlowJo software (TreeStar).

### *In vivo* depletion and blockade with monoclonal antibodies

CD25^+^ T cells were depleted with a single intravenous dose of anti-CD25 (PC61, 0.4 mg per mouse, BioXCell) on the day before second heart transplantation. Depletion was confirmed by loss of Foxp3^+^CD4^+^ cells as determined by flow cytometry of peripheral blood mononuclear cells 1 week post antibody administration.

### Statistical methods

The PS programme was used to calculate survival analyses^37^ and the online power calculator from Rollin Brant (University of British Columbia) was used for sample size calculations for comparison of two means. The two-tailed Student’s *t*-test or one- or two-way analysis of variance and *post hoc* Bonferroni test for multiple comparisons, or, where appropriate, non-parametric Mann–Whitney or Kruskal–Wallis with Dunn’s *post hoc* was performed to determine statistical differences between groups. Graft MST and *P* values were calculated using the Kaplan–Meier/log-rank test (Prism5; GraphPad Software Inc.).

## Additional information

**How to cite this article:** Miller, M. L. *et al.* Spontaneous restoration of transplantation tolerance after acute rejection. *Nat. Commun.* 6:7566 doi: 10.1038/ncomms8566 (2015).

## Supplementary Material

Supplementary Figures Supplementary Figures 1-2

Supplementary Movie 1 Spontaneous acceptance of a second donor-matched abdominal allograft next to the rejected first allograft by infected tolerant recipients. A first BALB/c heart was transplanted in the peritoneal cavity of a B6 recipient treated with anti-CD154 + DST resulting in transplantation tolerance. Infection with Lm i.p. on day 60 post-transplantation resulted in acute rejection of the first graft 1 week later. A second B/c heart was transplanted immediately below the first rejected allograft 3 weeks after Lm infection, in the absence of immunosuppression. The video was taken at sacrifice, 60 days after second transplantation, and shows the first non-beating white fibrotic allograft (white arrow) and the fully vascularized beating second donor-matched allograft (red arrow).
