# Supplementary material for: Spontaneous restoration of transplantation tolerance after acute rejection
Source: Nat Commun. 2015 Jul 7;6:7566. doi: 10.1038/ncomms8566 (PMC4498267; doi:10.1038/ncomms8566)
Supplement: Supplementary Figures — 1-2 [file ncomms8566-s1.pdf]

Supplementary Figure 1

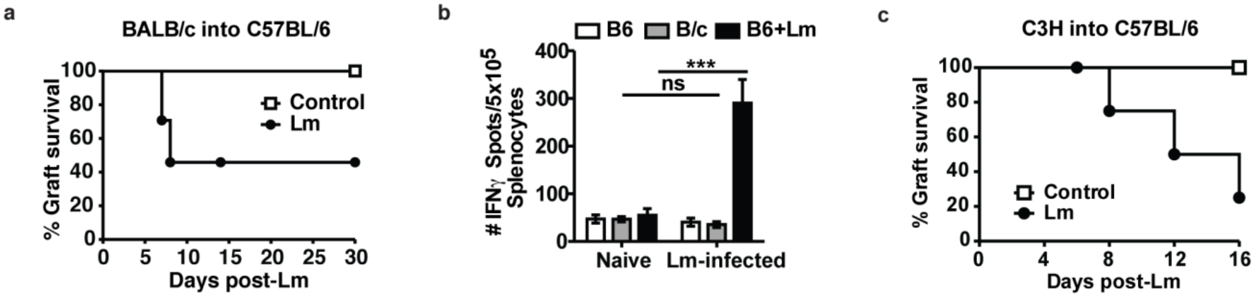

Supplementary Figure 1. Lm infection of transplant tolerant mice triggers acute allograft rejection in a subset of animals. BALB/c (**a**) or C3H/HEN (**c**) hearts were transplanted into B6 recipients treated with anti-CD154+DST at the time of transplantation (**a**, n=29; **c**, n=10). On day 60 post-transplantation, a subset of tolerant mice (**a**, n=24; **c**, n=5) was infected with Lm i.p. and graft survival was assessed in these mice and in uninfected control tolerant mice. $p<0.05$ by Log-rank test. (**b**) Naïve C57BL/6 mice were infected with Lm. Mice were sacrificed 6 weeks later and splenocytes from uninfected and Lm-infected mice were stimulated with T-depleted C57BL/6xBALB/c F1 (B/c) or syngeneic B6 splenocytes and analyzed by ELISpot (n=5 per group, experiment repeated twice). Data are presented as mean ± SEM. The syngeneic response was subtracted from the allogeneic response.

Supplementary Figure 2

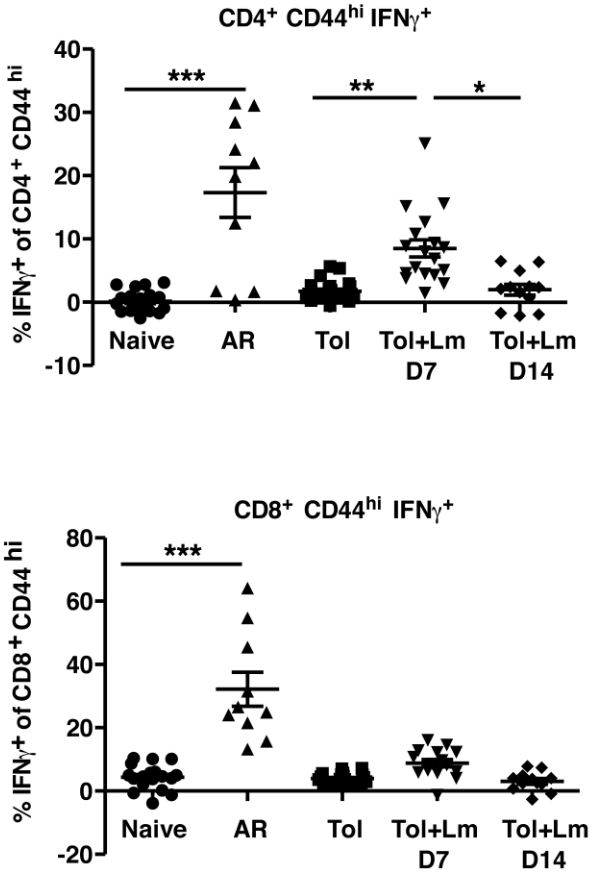

Supplementary Figure 2. Lm infection in tolerant transplant recipients results in transient increase in alloreactivity. BALB/c hearts were transplanted into B6 recipients either untreated (AR group) or treated with anti-CD154 at the time of transplantation (Tol group). Some tolerant animals were infected with Lm at day 60 post-transplantation (Tol+Lm group). Unmanipulated naïve mice were used as controls (naïve group, n=20). Animals were sacrificed 7 days after transplantation for the AR group (n=10), >60 days for the Tol group (n=20), and day 7 (n=15) or day 14 (n=12) for the Tol+Lm groups. The percentage of IFNγ^{+} CD44^{hi} CD4^{+} and CD8^{+} cells specific for alloantigen (%allo-%syn) is plotted. Results are pooled from five independent experiments and are presented as mean ± SEM. *p<0.05, **p<0.01, ***p<0.001 by One-way ANOVA with Bonferroni post-test.
